# Long‐term effects of single and combined introductions of antibiotics and bacteriophages on populations of *Pseudomonas aeruginosa*

**DOI:** 10.1111/eva.12364

**Published:** 2016-02-18

**Authors:** Clara Torres‐Barceló, Blaise Franzon, Marie Vasse, Michael E. Hochberg

**Affiliations:** ^1^ Institut des Sciences de l'EvolutionUniversité de MontpellierMontpellierFrance; ^2^ Santa Fe InstituteSanta FeNMUSA

**Keywords:** antibiotics, bacteriophage, combination therapies, evolution, *Pseudomonas aeruginosa*, resistance, virulence

## Abstract

With escalating resistance to antibiotics, there is an urgent need to develop alternative therapies against bacterial pathogens and pests. One of the most promising is the employment of bacteriophages (phages), which may be highly specific and evolve to counter antiphage resistance. Despite an increased understanding of how phages interact with bacteria, we know very little about how their interactions may be modified in antibiotic environments and, reciprocally, how phage may affect the evolution of antibiotic resistance. We experimentally evaluated the impacts of single and combined applications of antibiotics (different doses and different types) and phages on *in vitro* evolving populations of the opportunistic pathogen *Pseudomonas aeruginosa* PAO1. We also assessed the effects of past treatments on bacterial virulence *in vivo*, employing larvae of *Galleria mellonella* to survey the treatment consequences for the pathogen. We find a strong synergistic effect of combining antibiotics and phages on bacterial population density and in limiting their recovery rate. Our long‐term study establishes that antibiotic dose is important, but that effects are relatively insensitive to antibiotic type. From an applied perspective, our results indicate that phages can contribute to managing antibiotic resistance levels, with limited consequences for the evolution of bacterial virulence.

## Introduction

Despite widespread bacterial resistance and dwindling discovery of new molecules, antibiotics are still overwhelmingly the principal agent used against bacterial infections (Laxminarayan et al. 2013). Alternatives are needed and it has been argued that antimicrobial approaches and more generally chemotherapies often ignore insights coming from evolutionary biology (Read et al. 2011; Pena‐Miller et al. 2013). Specifically, study has shown that the goal of minimizing or eliminating pathogen populations through high‐dose therapies can be counterproductive, because it will select for resistant or refractory phenotypes (Hughes and Andersson 2012; Ramsayer et al. 2013), which will repopulate the infection, and potentially spread into the environment.

A promising alternative to antibiotics is the use of bacteriophages. Phage therapy employs highly specific isolates as one or more applications of potentially perpetuating, co‐evolving antibacterial agents (Viertel et al. 2014). The majority of assessments of phage therapy comes from observational study (Fruciano and Bourne 2007), and with a few notable exceptions (Bruttin and Brussow 2005; Wright et al. 2009; Sarker et al. 2012), most controlled experimental tests have been conducted *in vitro* or in animal models (Fu et al. 2010; Lood et al. 2015; Scanlan et al. 2015). Although some of these studies show support for the efficacy of phage therapy, one pervasive shortcoming is existing or evolved bacterial resistance (Bikard and Marraffini 2012; Seed et al. 2014).

Given the large population sizes attainable within a single bacterial infection (Leggett et al. 2012), it is not surprising that rare beneficial mutations have a higher probability of fixing, meaning that resistance evolution to single agents is a pervasive issue in control (Read et al. 2011; Gonzalez et al. 2013; Orr and Unckless 2014). Ecological and evolutionary theories provide testable hypotheses for when multiple control agents should be more effective at control than any subset (Hendry et al. 2011). Among alternatives to single agent, high‐dose therapies, increasing attention is being focused on combinations between two or more antibiotics (Hagihara et al. 2012), or antibiotics and phage (Lu and Collins 2009; Torres‐Barceló and Hochberg in press) as strategies for reducing or eliminating bacterial pathogen resistance. Given the potential diversity of bacteriophages predating a given bacterial strain (Kwan et al. 2005; Weitz et al. 2013), combinations of phages and antibiotics can be identified which will attack different bacterial targets and, should resistance mutations be present, these would be different for each agent thereby reducing the probability of the emergence of resistance mutations to either or both agents (Escobar‐Páramo et al. 2012; Hall et al. 2012).

Studies have demonstrated improved efficacy of associating phages and antibiotics to treat methicillin‐resistant *Staphylococcus aureus* (MRSA) (Kirby 2012; Chhibber et al. 2013), *Pseudomonas aeruginosa* (Hagens et al. 2006; Knezevic et al. 2013; Torres‐Barceló et al. 2014), and *Escherichia coli* strains (Ryan et al. 2012; Coulter et al. 2014). For example, Torres‐Barceló et al. (2014) recently showed that in sequential therapies against *P. aeruginosa*, the specific timing between phage and antibiotic introductions provides a window of opportunity for control. Some of these studies indicate a synergistic effect between both antimicrobial agents in preventing bacterial growth (Hagens et al. 2006; Kirby 2012; Knezevic et al. 2013; Torres‐Barceló et al. 2014), that is, that the effect of the combination is greater than the sum of effects produced by each agent separately (Loewe 1953). Whereas recent work suggests that double‐resistant bacteria would be strongly selected if using antibiotic cocktails (Pena‐Miller et al. 2013), antibiotic and phage combinations indicate the opposite effect (Verma et al. 2010; Kirby 2012; Zhang and Buckling 2012), although the underlying mechanisms remained unexplored. Moreover, little is known about the effects of antibiotic doses on the evolutionary process in combination therapies (e.g., for antibiotics and phages see Hagens et al. 2006; Torres‐Barceló et al. 2014). Also, with few exceptions (Kirby 2012), long‐term therapeutic effects, which may be more representative of *in vivo* situations, have not been explored. These two issues, dose and duration, are central in understanding resistance evolution (Read et al. 2011) and constitute a major challenge in employing combined therapies.

The continuous emergence of antibiotic resistance is especially important in Gram‐negative bacteria, which cause approximately 70% of the infections in intensive care units (Hernandez et al. 2013). The bacterium used in this study, *Pseudomonas aeruginosa*, is a leading cause of nosocomial infections and chronic lung infections in patients with cystic fibrosis (Mesaros et al. 2007). *P. aeruginosa* is intrinsically resistant to many antibiotics because of the limited permeability of its outer membrane and efflux pump systems (Breidenstein et al. 2011). It also has a high potential for resistance adaptation through mutational mechanisms, including increased efflux pump activity and enzymatic antibiotic modifications (Breidenstein et al. 2011). To date, antibiotic therapy is the principal means for controlling *P. aeruginosa* infections, and although combination therapies have been investigated involving multiple antibiotics (Traugott et al. 2011; Paul and Leibovici 2013), antibiotic‐phage associations have not been extensively investigated beyond the relative order of introduction of the antimicrobials (Escobar‐Páramo et al. 2012; Torres‐Barceló et al. 2014), and limited work has considered the effect of combining different concentrations of antibiotics or phages on bacterial virulence (Hosseinidoust et al. 2013). These are important questions, because it is not clear to what extent initially intense ecological interactions, longer‐term evolutionary effects, and/or their interactions influence outcomes.

In this study, we compare the effects of a range of doses of three antibiotics representing different modes of action on *P. aeruginosa* populations in the presence or absence of phages. With the aim of assessing long‐term efficacy, we exposed bacteria to the different treatments for 7 days. We evaluated important aspects of bacterial evolutionary potential such as adaptation rate, final density, antibiotic resistance, and resistance to phage, using *in vitro* assays. Given the possibility that treatment does not clear the pathogen and affects the severity of an infection, we also measured the consequences of treatments for bacterial virulence *in vivo* using wax moth larvae. We find that for all antibiotics tested, phages and antibiotics have synergistic effects on reducing bacterial populations, and this is positively correlated with antibiotic dose. Even when single and combined treatments had similar effects on reducing bacterial densities, recovery rates were slower for all the combined antibiotic‐phage conditions. Interestingly, combination treatments limited antibiotic resistance levels compared to antibiotic treatments alone, whereas antibiotics did not have this reverse effect on phage resistance. Finally, both single and combined treatments reduced bacterial virulence in wax moth larval hosts compared to untreated ones, but phage‐treated bacteria attenuated the magnitude of the reduction in virulence. We discuss the relevance of our findings for future research aimed at treating bacterial infections in human health care.

## Materials and methods

### Bacteria and phages

We used the bacterium *Pseudomonas aeruginosa* PAO1 (F.E. Romesberg's strain, Cirz et al. 2006) and the lytic phage LKD16, from the Podoviridae family (Ceyssens et al. 2006). All experiments were carried out in 96‐well plates, with bacteria growing in 200 μL of King's B (KB) medium at 37°C without agitation. M9 medium was used for dilutions. The phage stock was prepared as described elsewhere (Betts et al. 2013). Briefly, 10% vol/vol chloroform was added to phage‐containing bacterial cultures, vortexed, and centrifuged. Phage‐containing supernatants were carefully recovered and stored at 4°C. This LKD16 stock was tittered (1.12 × 10^7^ PFU/μL) and used as the ancestral phage for all the experiments.

### Treatments and experimental evolution

Individual clones of *P. aeruginosa* PAO1 were isolated in KB solid agar plates from the clonal stock stored at −80°C. Three microcosms with 6 mL of KB were used to each amplify a different, arbitrarily selected clone under continuous shaking (200 rpm) for 6 h to obtain an exponential phase culture of *c* 10^6^ cells/mL. These microcosms were then mixed and used as the inoculum for the evolution experiment.

To study the long‐term effects of different phage‐antibiotic treatments, *c* 2 × 10^3^ cells of *P. aeruginosa* PAO1 (from a mix of the three microcosms) were inoculated into fresh media containing phage‐only, antibiotic‐only, or phage‐antibiotic conditions. We used the antibiotics carbenicillin, gentamicin, and trimethoprim (Sigma‐Aldrich, St. Louis, MO, USA), belonging to the following families (bacterial pathways targeted): *β*‐lactam (inhibits cell wall synthesis); aminoglycoside (blocks protein synthesis); sulfamide (interferes with nucleic acid synthesis), respectively. These antibiotics were added to liquid medium at the required concentrations to inhibit 5%, 50%, and 95% of bacterial growth, hereafter referred to as IC5, IC50, and IC95, respectively (see Table 1 for the specific concentrations). Phage dose was determined in pilot studies as that achieving 90% bacterial growth inhibition relative to a control after 24 h (1 PFU/100 bacteria). This concentration of a moderately virulent phage allowed us to follow bacterial population dynamics under conditions where both bacterial extinction and complete resistance were unlikely.

**Table 1:** Concentrations of the different antibiotics for ID5, ID50, ID95, and MIC, in mg/L

	Antibiotic concentrations (mg/L)			
	IC5	IC50	IC95	MIC
Carbenicillin	9	42	67	102
Gentamicin	27	42	61	70
Trimethoprim	64	162	444	512

Six replicates of each treatment were distributed arbitrarily in three 96‐well plates, for a total of 108 populations [six replicates × three antibiotics × three doses × two (phage presence or absence)]. An equivalent number of replicates per plate with phage‐only treatments and nontreated bacteria (positive controls) were also established (six replicates × two controls × three plates: 36 populations). The total number of populations was 144. Negative controls with media only were distributed in all plates to monitor for the occurrence of possible contamination.

Bacterial density was measured every 24 h by means of optical density (OD) at 600 nm (Fluostar, BMG LAB‐TECH, Ortenberg, Germany). So as to homogenize cultures, thorough pipette mixing of each well was performed before transferring each population into a new well containing either fresh media under the same treatment conditions (antibiotics and/or ancestral phages) or under control conditions. A volume corresponding to 10% of each population was calculated and transferred, to minimize possible bottlenecks. This same protocol was carried out for seven consecutive days. After being transferred, the remainder of the microcosm was stored in 20% glycerol at −80°C for further analysis.

### Phage effect on bacterial growth

To measure bacterial resistance to phage, we compared the growth of either evolved or ancestral bacteria, with or without ancestral phage. Final bacterial populations and the ancestral stock, all stored at −80°C, were streaked on solid agar KB plates to isolate colonies. Four replicate populations of each treatment were used, and one colony of each was amplified in 1 mL of KB in 48‐well plates at 37°C. After overnight incubation, all cultures were centrifuged at 20 000 *g*, the supernatant discarded, and the pellet resuspended in M9. This procedure eliminated most previous phage present in the evolved bacteria. OD was then standardized to the same level for all tested populations, *c* 10^6^ cells added to 200 μL of KB with and without the ancestral LKD16 phage (1 PFU/100 bacteria), and OD recorded every 20 min for 24 h at 37°C. To minimize the effects of condensation and biofilm interference on OD readings, 10 s of agitation at 200 rpm was programmed to occur before each measurement.

### Determination of antibiotic resistance levels

Antibiotic resistance level was measured as the minimum inhibitory concentration (MIC) at which no bacterial growth was detected. Approximately *c* 2 × 10^3^ cells of each final treatment population were inoculated into 200 μL of media containing concentrations of each respective antibiotic at twofold increments. After 24 h, we scored growth inhibition as OD < 0.1. Antibiotic resistance was scored for the evolved populations relative to that of the ancestor (normalized to MIC = 1). Thus, a value of two reflects a doubling in the MIC relative to the ancestral clone, three is twice the MIC of two, and four twice the MIC of three.

### Virulence assays

The virulence of evolved bacteria and ancestral clones was measured by inoculating a sample from each of the frozen final populations and the frozen ancestral stock, respectively, into fresh media and incubating at 37°C until obtaining exponential phase cultures (OD between 0.4 and 0.7). Bacterial replicates from the same treatment were then pooled, centrifuged, and resuspended in M9 buffer and the density adjusted through dilution to *c* 5 × 10^3^ cells/mL. 20 μL of each bacterial treatment was injected into each of 12 wax moth (*Galleria mellonella*) larvae of homogeneous size (*c* 2–3 cm) with a micro‐injector. Negative control treatments injecting only M9 were also performed. Larvae were kept at 37°C. The virulence of evolved and ancestral bacteria was assessed as mortality rate per treatment, established by observing larval mortality every hour for 48 h.

### Statistical analysis

All statistical analyses were conducted using R statistical software (R 3.1.1; http://www.r-project.org/). For the main analysis, we applied fully factorial models by including phage treatment (yes/no), antibiotic type, and antibiotic dose as explanatory factors. Repeated measures through time were included as random factors when present. Where appropriate, analyses were carried out separately for single and combined treatments.

Specifically, we performed linear mixed‐effects models for the analysis of bacterial density through time and *t*‐tests to compare densities between treatments for final (day 7) densities. To calculate the rate of adaptation over the seven days of the experiment, a nonparametric and nonlinear approach was carried out as follows. First, the deviation from the mean OD of the untreated control of each plate at each time (which varied significantly between plates and in time *F*_4,158_ = 1181.731, *P* < 0.001) was subtracted from the observed OD values to correct for ‘plate’ and ‘time’ effects. In a second step, a model was fitted for each replicate via a logistic‐type nonlinear least squares function (Kahm et al. 2010):

$$y\left(t\right)=\frac{A}{1+e^{\left(\frac{4\mu }{A}\left(\lambda -t\right)+2\right)}}$$

A smoothed cubic spline was used to estimate the lag phase *λ*, the maximum slope μ, and maximum growth *A* (‘grofit’ package, Bates and Chambers 1992). Once statistically fit, we calculated the rate of adaptation (*r*) from the following equation:

$$r=\partial_{t}\text{log}\left[y\left(t\right)\right]=\frac{4{\left(1-\frac{1}{1+e^{\left(\frac{4\mu \lambda }{A}+2\right)}}\right)}^{\mu }}{A}$$

and used anovas to analyze the effect of the different treatments on *r*.

We investigated whether combined treatments were subadditive, additive, or superadditive (i.e., synergistic) by pairing random couples of single‐phage with single‐antibiotic replicates at each time‐point of the evolution experiment for each dose and antibiotic type. For both replicates in a pair, we calculated the reduction in bacterial density relative to the untreated controls. We then added the two single treatment reductions to obtain the expected density in a hypothetical combined treatment. We performed this operation for every replicate in the experiment. The effect was scored as synergistic if ΔOD_phage_ + ΔOD_ab_ < ΔOD_phage+ab_, where Δ is the absolute decrease in optical density (OD), and phage+ab is the combined treatment. The maximum additive effects were limited to the untreated control OD values, the biological meaningful threshold (i.e., maximum reduction is the density of the untreated control). We then compared densities between observed and expected replicates in a factorial model. A nonparametric Spearman correlation was used to test the linear dependence between antibiotic dose and synergy (calculated as observed values subtracted from expected values) variables.

We employed General Linear Model techniques to analyze variation in antibiotic resistance levels (MIC). To study the possibly inhibitory effect of ancestral phages in the final populations, we considered growth over 24 h and the final OD after 24 h. The area under the curve (AUC) was estimated for each OD dynamic with the R package ‘MESS’ (Ekstrom 2011). The estimated AUCs were compared between treatments using anovas. Multinomial analyses were used to analyze the effect of treatments on the final OD.

Time‐dependent larval survival was estimated using the Kaplan–Meier technique (Bland and Altman 1998). A multivariate Cox model was then applied to compare the survival curves due to bacteria isolated from different treatments.

## Results

### Analysis of bacterial population dynamics over 7 days

**Figure 1:**
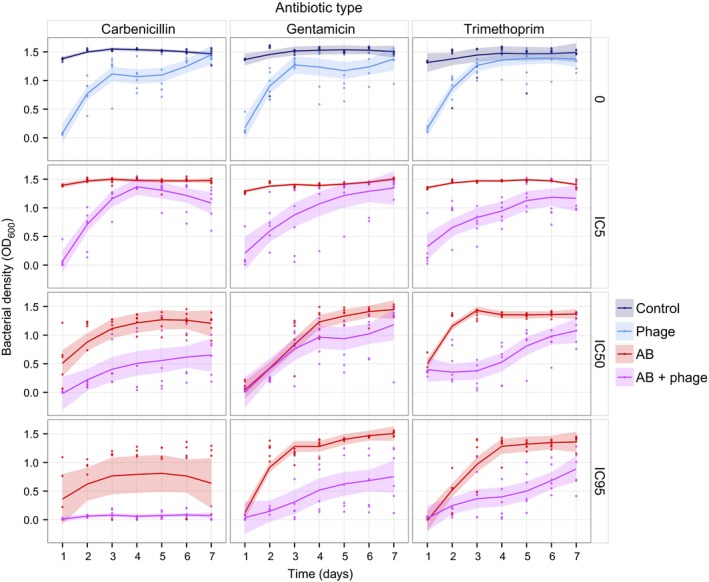
Bacterial density dynamics measured every 24 h for 7 days. The horizontal panels represent the different concentrations of antibiotics relative to the resulting % inhibition of growth (IC: inhibitory concentration), and the vertical panels represent the different antibiotic types used. ‘Control’ corresponds to untreated bacteria, ‘phage’ to phage only, ‘AB’ to antibiotic only, and ‘AB + phage’ to combined phage and antibiotic treatments. Solid lines represent the means of eight populations. Shaded regions are 95% confidence intervals.

As expected, the addition of phage or antibiotic had a strong negative effect on bacterial densities during the experiment (Linear mixed‐effect Model, phage: *F*_1,42_ = 340.19, *P* < 0.0001; antibiotic: *F*_1,42_ = 143.85, *P* < 0.0001; Fig. 1). Across time, there were significant antibiotic type and dose effects and interactions between these and the addition of phage (LME, antibiotic type: *F*_2,709_ = 19.90, *P* < 0.0001; antibiotic dose: *F*_1,32_ = 184.68, *P* < 0.0001; phage x antibiotic type: *F*_2,709_ = 2.29, *P* = 0.0445; phage x antibiotic dose: *F*_1,32_ = 1.91, *P* = 0.0061; Fig. 1), meaning that the specific combinations of different antibiotics with phage had an impact on bacteria density dynamics. At final bacterial densities (day 7) however, the effect of antibiotic type disappears and only the addition of phage and antibiotic dose are significant (LME, antibiotic type: *F*_2,66_ = 28.93, *P* = 0.2530; phage: *F*_1,32_ = 60.97, *P* = 0.0139; antibiotic dose: *F*_1,32_ = 55.23, *P* < 0.0001; Fig. S1). Specifically, bacteria treated with phage and antibiotics reached an average final optical density (OD) of 0.92 ± 0.07, whereas antibiotic‐only treated bacteria had an OD of 1.32 ± 0.05. Final density values showed a significant negative correlation with antibiotic dose (Pearson's test: *t* = −6.92, d.f. = 142, *P* < 0.0001). These results indicate that the addition of phage combined with high doses of any of the antibiotics reduces the long‐term growth of *P. aeruginosa* PAO1.

We find that bacterial density recovery rates (slopes of bacterial density over 7 days of treatment) are marginally affected by the addition of phages and significantly by antibiotics (anova:* F*_1,134_ = 3.27, *P* = 0.0660; *F*_2,134_ = 13.80, *P* = 0.0071, respectively). On average, slopes calculated with a nonparametric and nonlinear approach (see Material and Methods) are higher and increase with antibiotic dose (*F*_1,48_ = 29.45, *P* < 0.0001) under antibiotic‐only treatments (2.08 ± 0.36) (Left caption, Fig. S2). In contrast, in the double treatments we observe smaller recovery rates (1.85 ± 0.22), higher at the lowest antibiotic doses, and decreasing as the concentration of antibiotic increases, indicating a significant interaction between the addition of phages and antibiotic dose (anova antibiotic dose: *F*_1,48_ = 11.73, *P* = 0.0013; antibiotic dose x phage: *F*_1,98_ = 39.13, *P* < 0.0001; Right caption, Fig. S2). These results thus show a pronounced effect of high antibiotic dose when combined with phages in limiting bacterial population recovery, whereas bacteria recover faster with high dose of antibiotics alone. Furthermore, we detect significant effects of using different antibiotic types in single treatments, but not in the presence of phages (anova antibiotic type: *F*_2,48_ = 6.40, *P* = 0.0034; *F*_2,48_ = 1.87, *P* = 0.1659, respectively).

**Figure 2:**
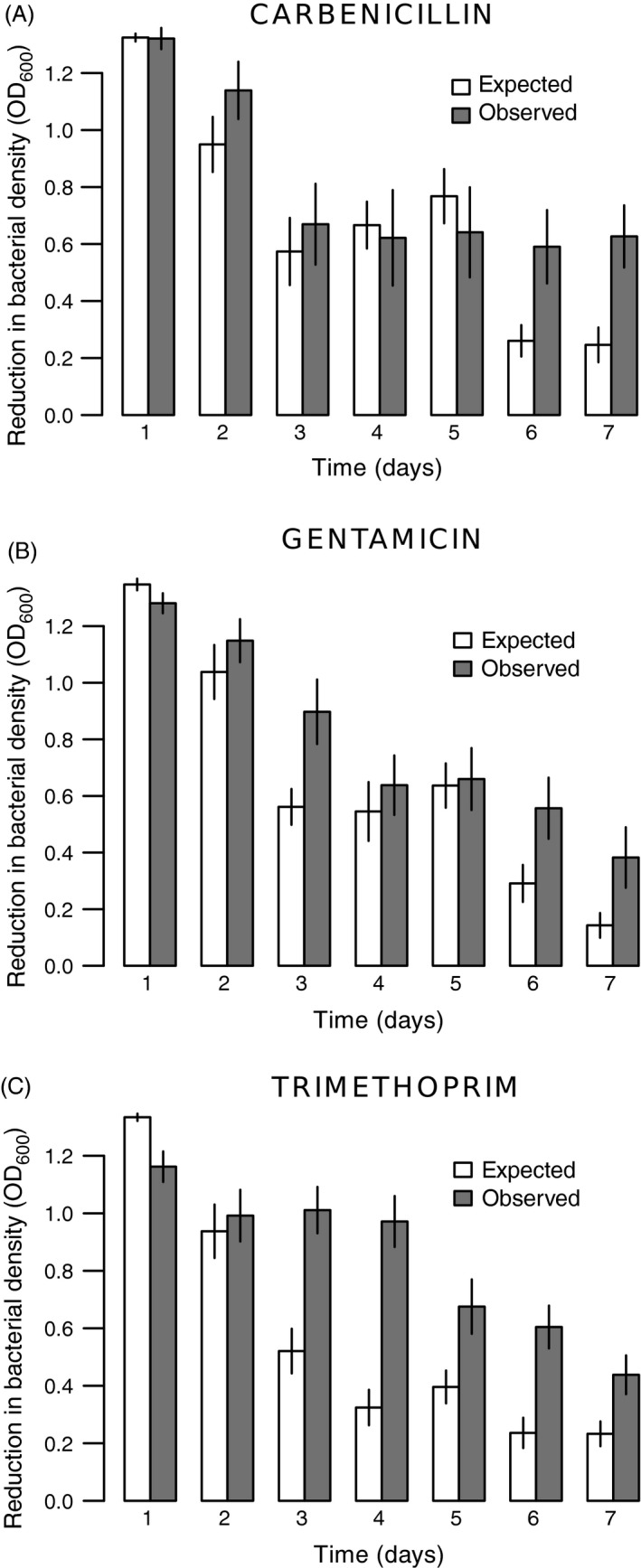
Synergistic effects of treatments through time depending on antibiotic type (Panels (A) carbenicillin; (B) gentamicin; (C) trimethoprim). Expected additive versus observed effects of combined phage‐antibiotic treatments in preventing growth in bacterial populations. Effects on growth are measured as decreases in density relative to the control. A synergistic effect was identified when the observed OD difference was significantly higher than the expected additive effect. The expected additive effect was calculated by summing the individual effects of phage and antibiotic treatments. Different antibiotic doses were grouped. Bars represent standard errors.

**Figure 3:**
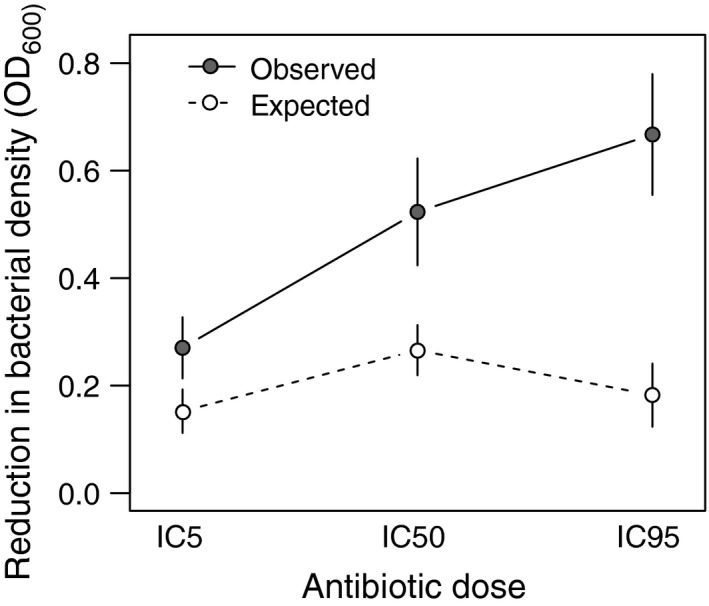
Synergistic effects in final bacterial populations (day 7) as a function of antibiotic dose (% inhibition of growth IC). Expected additive versus observed effects of combined phage‐antibiotic treatments in preventing growth in bacterial populations. Effects on growth are measured as decreases in density relative to the control. The expected additive effect was calculated by summing the individual effects of phage and antibiotic treatments. A synergistic effect was identified when the observed OD difference was significantly higher than the expected additive effect. Antibiotic types were grouped. Bars represent standard errors.

A synergistic effect was identified when the recorded combination OD difference (observed) was significantly higher than the expected additive effect (see Material and Methods for more details). Overall, the combination of phages and antibiotics had a synergistic effect on reductions in bacterial density (LME: *F*_1,34_ = 58.71, *P* < 0.0001; Fig. S3). However, we see no such effect over the first 2 days of the experiment (LME: *F*_1,34_ = 0.08, *P* = 0.7839; Figs 2, S3), presumably because of the initial strong ecological effect of each antimicrobial separately. Beyond two days, the combination of phages and antibiotics resulted in a considerable extra (i.e., synergistic) decrease in the populations compared to a simple additive effect (LME: *F*_1,34_ = 74.18, *P* < 0.0001; Fig. S3). There were significant differences between antibiotic types in the synergistic effect through time (LME: *F*_2,34_ =19.69, *P* < 0.0001; Fig. 2). Nonetheless, analogous to the density results, the antibiotic type effect vanishes by the end of the experiment (anova:* F*_2,34_ = 3.09, *P* = 0.0507), whereas the antibiotic dose component plays an important role for the duration of the experiment and at the final time‐point (LME: *F*_1,34_ = 144.74, *P* < 0.0001; day 7 anova:* F*_1,34_ = 16.11, *P* = 0.0001; Fig. 3). Indeed, there is a strong positive correlation between antibiotic dose and the synergistic effect (Pearson's correlation: *t *= −5.24, d.f. = 334, *P* < 0.0001; Fig. 3). Comparing the final observed and expected effects, carbenicillin produced the maximum synergistic effect, with a threefold difference between expected and observed values in the reduction of bacterial density, compared to the twofold difference observed for gentamicin and trimethoprim (Fig. 2).

In summary, bacterial densities are significantly affected by the combination of phages and antibiotics compared to either separately. The higher the dose of antibiotic combined with phage, the more populations decreased and the slower they recovered. In contrast, with antibiotics only, the higher the dose, the faster the rate at which bacteria recovered. This is also reflected in the combined effects of antibiotics and phages being synergistic and dependent on the antibiotic dose used.

The synergistic effect observed between antibiotic and phages suggests that either double‐treated bacteria were not capable of achieving complete resistance to phages or antibiotics, or that the surviving (resistant) populations carried costs that impaired their normal growth. To investigate these possibilities, we measured: (i) ancestral phage effects on bacterial growth capacity, (ii) antibiotic resistance levels, and (iii) growth capacity of the final bacterial populations.

### Analysis of final treated bacteria

#### Resistance to phages

**Figure 4:**
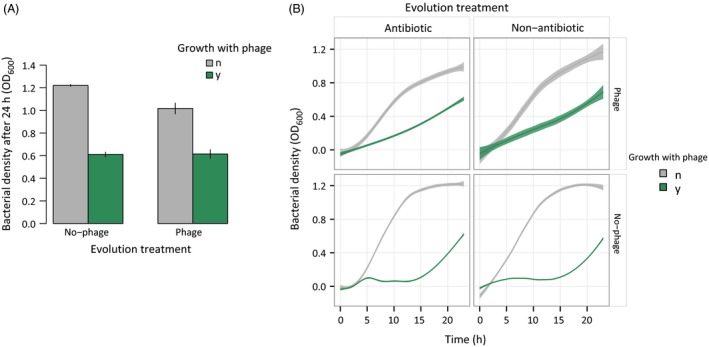
Effects of reintroducing ancestral phage into final bacterial populations. Panel (A) represents the final OD attained by bacteria in 24 h depending on if they were evolved with or without phages (*x*‐axis). The legend indicates re‐exposure or not to the ancestral phage (green and gray, respectively). Bars are standard errors. Panel (B) shows the OD values over 24 h used to determine the AUC (area under the curve) in the presence or absence of ancestral phage (green and gray, respectively). The different vertical panels represent treatments of bacteria evolved with antibiotics or not. Horizontal panels divide treatments where phage was or was not introduced as a treatment. Antibiotic types and doses were grouped. Shaded regions are 95% confidence intervals.

To estimate the effect of previous treatment on bacterial growth in the presence of ancestral phages, we isolated colonies from the end of the experiment (day 7) and inoculated them with the ancestral phage. We then measured final densities after 24 h to assess the inhibitory effect of phages and also the area under the curve (AUC) as an estimate of the overall bacterial growth capacity (Fig. 4).

We observed a strong effect of ancestral phage in decreasing bacterial densities after 24 h (1.12 ± 0.03 reduced to 0.62 ± 0.02; anova:* F*_1,70_ = 52.13, *P* < 0.0001). Surprisingly, this reduction was not significantly different between bacterial populations previously treated with or without phage (anova:* F*_1,75_ = 0.019, *P* = 0.8920; Fig. 4A). The final bacterial density in the presence of ancestral phage did not differ depending on previous treatment with different antibiotic types or doses (anova, antibiotic type effect: *F*_2,75_ = 1.10, *P* = 0.364; antibiotic dose: *F*_1,75_ = 0.02, *P* = 0.881). These results suggest that, independent of treatment, evolved bacteria were still highly susceptible to the ancestral phage.

A deeper analysis of the effect of the ancestral phage on overall 24 h bacterial growth measured as the AUC (area under the curve) revealed slightly different effects. When confronted with ancestral phage, bacteria that had been previously treated with phage showed a linear increase and higher AUC values than non‐phage‐treated bacteria, the latter exhibiting an initial lag phase with a small peak, followed by exponential recovery (anova:* F*_1,60_ = 9.32, *P* = 0.0034; Fig. 4B). Nonetheless, single‐antibiotic treatments or combined treatments with phage did not differ in overall growth with ancestral phages (*F*_1,80_ = 1.08, *P* = 0.302). In single treatments, neither antibiotic type nor dose significantly affected the AUC in the presence of ancestral phage (*F*_2,60_ = 1.55, *P* = 0.2200; *F*_1,60_ = 0.09, *P* = 0.7720, respectively). In contrast, antibiotic type was found to be significant in combined treatments, whereas there was no effect of antibiotic dose (*F*_2,20_ = 5.06, *P* = 0.0093; *F*_1,20_ = 0.04, *P* = 0.8524, respectively). However, when combined and only‐phage treatments were analyzed separately, none of the three antibiotics had a significant effect on phage resistance (carbenicillin, *F*_1,20_ = 0.48, *P* = 0.4907; gentamicin, *F*_1,20_ = 0.18, *P* = 0.6757; trimethoprim, *F*_1,20_ = 3.49, *P* = 0.0618). These results show that treatments with and without phages have different growth dynamics in the presence of the ancestral phage, even though the final outcome (final OD) was apparently unchanged, indicating the expression of partial resistance.

Finally, as expected in the absence of ancestral phage, the growth capacity was significantly limited for bacteria resulting from combined phage‐antibiotic treatments compared to either single treatment, both for final OD and the AUC (*F*_1,75_ = 21.47, *P* < 0.0001; *F*_1,60_ = 23.60, *P* < 0.0001, respectively, Fig. 4). This difference suggests a cost of resistance in double treatments, constraining them to suboptimal growth even in the absence of antimicrobials.

#### Resistance to antibiotics

**Figure 5:**
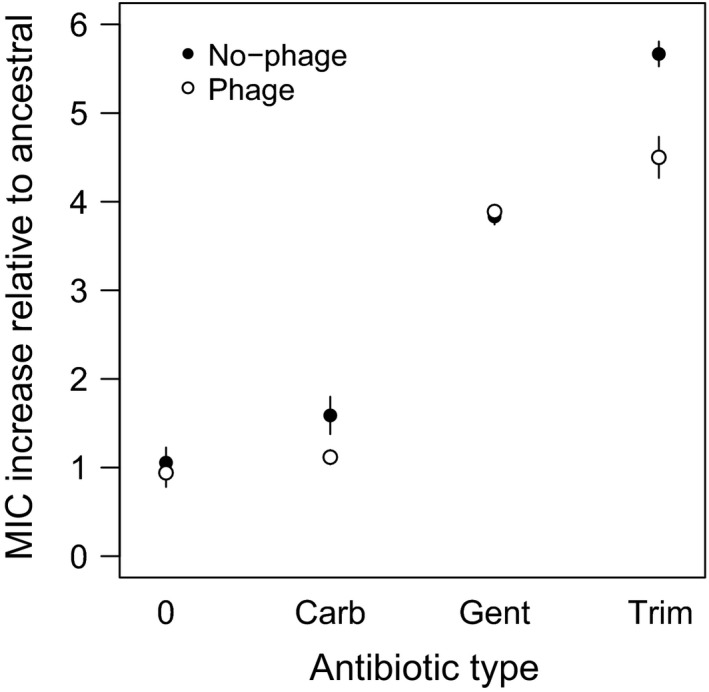
Antibiotic resistance increases in final bacterial populations relative to the ancestor (assigned as MIC = 1) regarding the presence and type of antibiotic. ‘No‐phage’ and ‘phage’ indicate whether bacteria evolved with phages. Antibiotic types are assigned as ‘0’ for nonantibiotic treatments, ‘Carb’ for carbenicillin, ‘Gent’ for gentamicin, and ‘Trim’ for trimethoprim. Different antibiotic doses were grouped. Bars represent standard errors.

We then tested whether the combined treatments limited the final level of antibiotic resistance (MIC) acquired by the surviving evolved bacteria compared to ancestral levels. We also determined the magnitude of the change in antibiotic resistance generated by the different antibiotic doses and types. Treatments with antibiotics resulted in different levels of increase in the MIC compared to controls, but the addition of phages attenuated this effect (multinomial analysis, antibiotic addition; *χ* ^2^ = 13.01, d.f. = 5, *P* = 0.0233; phage addition: *χ* ^2^ = 22.482, d.f. = 5, *P* = 0.0004). We found evidence for an antibiotic type effect, but not an antibiotic dose effect on the MIC increase relative to the ancestral bacteria (MNA, antibiotic type: *χ* ^2^ = 192.56, d.f. = 10, *P* < 0.0001; Fig. 5; antibiotic dose: *χ* ^2^ = 7.01, d.f. = 5, *P* = 0.2201). These results suggest that phages constrain the evolution of antibiotic resistance in combined treatments.

#### Virulence

**Figure 6:**
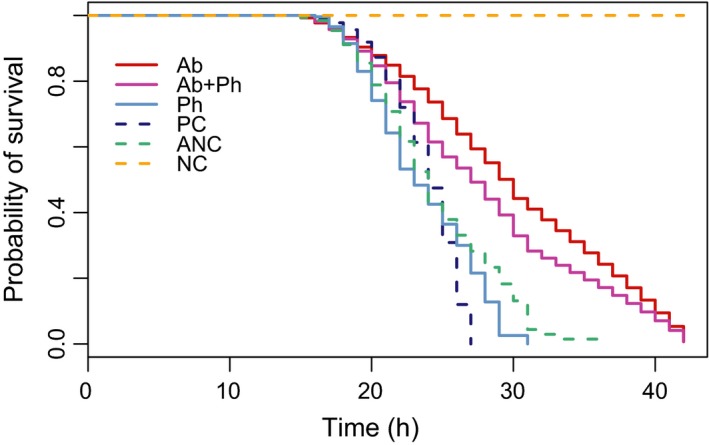
Probability of survival of *Galleria mellonella* larvae inoculated with ancestral or final bacterial populations from the different treatments. Antibiotic doses and types are grouped. Ab (only antibiotics), Ab+Ph (antibiotics and phages), Ph (only phages), PC (evolved without any treatment), ANC (ancestral bacteria), NC (larvae inoculated with buffer only).

We then analyzed the consequences of the 7‐day treatments on bacterial virulence *in vivo*, inoculating the evolved bacteria into wax moth larvae (*Galleria mellonella*) and measuring their life span. Supplementing phages in addition to antibiotics daily *in vitro* reduced *P. aeruginosa* final virulence in wax moth larvae compared to nontreated controls, but the reduction was smaller than that of bacteria treated with antibiotics alone (combined treatments versus controls: *Z* = −15.7, d.f. = 3, *P* < 0.0001; combined treatments versus antibiotics‐only: *Z* = 4.89, d.f. = 5, *P* < 0.0001; Fig. 6). Phage‐only treatments produced bacteria that were on average 17% more virulent than the nontreated controls (*Z* = 2.76, d.f. = 1, *P* = 0.0057), but still significantly less virulent than ancestral bacteria (*Z* = −5.46, d.f. = 1, *P* < 0.0001). In general, all evolved bacteria (controls and treatments) were less virulent relative to ancestral bacteria (nontreated versus ancestral: *Z* = −4.90, d.f. = 1, *P* < 0.0001), and we found statistically significant differences depending on the antibiotic type and dose used (*Z* for type = 10.31, d.f. = 2, *P* < 0.0001; for dose: *Z* = 12.86, d.f. = 2, *P* < 0.0001, Fig. S4), with low doses reducing virulence compared to higher doses.

## Discussion

Previous study has considered the separate effects of phages and antibiotics on bacterial population sizes and resistance (Hall et al. 2012; Pena‐Miller et al. 2013), and several recent investigations have assessed simultaneous and/or sequential applications of these antimicrobials (Escobar‐Páramo et al. 2012; Hagihara et al. 2012; Zhang and Buckling 2012; Knezevic et al. 2013; Torres‐Barceló et al. 2014; for recent review, see Torres‐Barceló and Hochberg in press). Whereas theory indicates that other mitigating factors may be important in the evolutionary process, specifically the force of selection (Holt and Hochberg 1997) and interactions between control agents (Kim et al. 2014), these basic theoretical predictions have not been empirically tested. In this work, we determined the short‐ and long‐term impacts of either phages and/or antibiotics (three different types at three different doses) on populations of the pathogenic bacterium *P. aeruginosa*.

Our findings confirm previous study showing that phage‐antibiotic combinations typically result in larger reductions of bacterial populations and the same or less resistance than either antimicrobial introduced in isolation (Escobar‐Páramo et al. 2012; Zhang and Buckling 2012; Torres‐Barceló et al. 2014). Nevertheless, we found that certain factors may mitigate the selective impact of antibiotics on bacterial populations. Our results can be summarized as follows. First, increasing antibiotic dose resulted in greater reductions in bacterial population densities and adaptive potential. Second, at least for the antibiotic types we tested that are associated with different bacterial targets, significant synergistic effects with phage were observed. Third, we show that *in vitro* antibiotic resistance levels are significantly reduced by the presence of phages, generalizing previous results (Verma et al. 2010; Kirby 2012; Zhang and Buckling 2012) to other antibiotic classes. Finally, we found that bacterial virulence is generally reduced by the treatments, but the effects of antibiotics are significantly greater than phage. We discuss these and other findings in more detail below.

Evolutionary theory suggests that, all else being equal, higher doses and longer exposure times to chemotherapeutic agents should increase selection pressure for resistance (Read et al. 2011), but empirical study shows that many mitigating factors may reduce or reverse this prediction (Kouyos et al. 2014). One important factor is the probability of obtaining resistance mutations associated with different costs and study has shown, for example, how low‐dose strategies may increase the fixation of low cost resistance mutations, partially compromising therapeutic outcome (Kouyos et al. 2014). In the present work, higher doses of antibiotic‐only treatments for three different antibiotics lead to more rapid bacterial recovery (Fig. S2). A recent study found a similar effect (i.e., ‘evolutionary rescue’) when testing different doses of streptomycin with *P. fluorescens*, possibly due to different emergence times or different relative benefits of favorable mutations (Ramsayer et al. 2013). Conversely, when combined with a lytic phage, we find that the higher the antibiotic dose, the slower the bacteria recover in terms of population density (Fig. S2). As the initial effect of reducing population densities did not differ significantly for the highest doses of single and combined treatments, it would appear that the initial probability that a population generates beneficial mutations cannot explain this result. More likely explanations include mechanistic trade‐offs between double resistance to antibiotics and phages, and costly compensatory mutations that would be less readily fixed under these conditions compared to the single antimicrobial treatments. This last hypothesis is supported by the lower growing capacity recorded in the absence of the phage and antibiotic for the double treatments compared to single antimicrobial conditions (Fig. 4).

A second conclusion from our study is that synergistic effects with this particular phage are observed for all three antibiotics (Fig. 2), indicating some level of generality across their different targeted mechanisms of action. It has been suggested (Zak and Kradolfer 1979) that the different cellular morphologies induced by these antibiotics play a role in interactions with phages leading to synergy. Gentamicin induces cell enlargement, and carbenicillin and trimethoprim cause elongation and filamentation (Zak and Kradolfer 1979). These phenotypes have been associated with increased rate of phage production, probably due to the altered physiological state and sensitivity to lysis (Comeau et al. 2007). However, we found that carbenicillin was particularly synergistic in combination with phages, suggesting that cell wall disruption is somehow involved in the enhancement of phage predation. Perturbations in the peptidoglycan layer produced by low doses of ß‐lactam antibiotics have been suggested to not only alter cell morphology, but also to accelerate cell lysis produced by different phages in *E. coli* (Comeau et al. 2007). We do not know to what extent, however, such a mechanism may be acting in our system, involving a different microbial host and higher antibiotic concentrations. Moreover, our results indicate that for all three antibiotics, synergy was not observed during the initial phases of the interactions, but rather only at the end of the experiment (Fig. S3), suggesting that it was associated with bacterial evolution. One possible explanation is that during the first hours of contact, both single and double treatments considerably decrease bacterial density, whereas after six serial transfers (*c* 50 bacterial generations), simultaneous resistance to antibiotics and phages entails larger costs than to either mortality agent separately. The net result is lower than expected populations in the combined treatments, but only once costly resistance emerges as a result of selection and evolution. Consistent with this explanation, several studies have shown that phages limit the emergence of antibiotic resistant variants in combined treatments (Verma et al. 2010; Escobar‐Páramo et al. 2012; Kirby 2012; Zhang and Buckling 2012; Torres‐Barceló et al. 2014). Additional research is necessary, however, to understand how and why relative times of introduction affect this phenomenon.

Third, whereas *in vitro* antibiotic resistance levels are significantly reduced by the presence of phages, the corresponding resistance to phages did not differ between treatments in the presence or absence of antibiotics (Fig. 4). Betts et al. (2013) previously showed that when bacteriophage LKD16 was passaged on *P. aeruginosa*, phage resistance levels attained *c* 80% after six transfers, and subsequent study indicated that resistance to this phage is costly, because it is partially lost when bacteria and phages coevolved (Betts et al. 2014). Different constraints on the independent evolution of antibiotic and phage resistances could explain why LKD16 phages affected antibiotic resistance levels but not vice versa, suggestive of different pleiotropic effects. Further research is needed to investigate the mechanisms behind this asymmetric effect.

Finally, past studies have found different relationships between elevated antibiotic resistance and virulence expression, the most frequent being negative (Beceiro et al. 2013). For example, the loss of porins increases antibiotic resistance but decreases virulence in many bacterial species, whereas effects in bacterial strains showing upregulation of efflux pumps and virulence are less clear (Beceiro et al. 2013). On the other hand, while lysogenic phages can transfer virulence factors (Fortier and Sekulovic 2013), the majority of previous studies show that bacteria resistant to lytic phages have attenuated virulence on the host, whether arthropods, fish, plants, mice, or humans (Evans et al. 2010; Filippov et al. 2011; Hall et al. 2012; Laanto et al. 2012; Seed et al. 2014). Costly resistance to phages and/or antibiotics can impact relevant pathogenicity factors and/or lessen bacterial growth rates, resulting in less harm to the host (Beceiro et al. 2013; Seed et al. 2014). Our results showing that antibiotics alone reduce virulence are in line with most previous experimental studies, but we find that LKD16 phages lower virulence less than antibiotics (Evans et al. 2010; Laanto et al. 2012). Specifically, we found that *in vivo* inoculation of wax moth larvae with carbenicillin‐treated bacteria resulted in the least virulent bacteria (Fig. S2), possibly due to resistance mutations in the peptidoglycan synthesis pathway, this being implicated in bacterial pathogenesis (Godlewska et al. 2009). Comparing the doses applied, the weakest dose of antibiotics (IC5) of single‐antibiotic treatments decreased the time to death of the larvae significantly more than higher dose treatments. Because this effect was not significant for all antibiotic types, this suggests specificity in either the action or resistance mechanisms associated with gentamicin and trimethoprim. For example, Haddadin et al. (2010) showed that some antibiotics at sub‐MIC levels interfere with bacterial biofilm virulence expression. Nevertheless, our results suggest that combined therapies do not result in the largest reduction in virulence and that antibiotic dose produces a counterintuitive effect, with low doses reducing virulence more than higher ones. Consistent with this effect, it has been demonstrated that sub‐MIC levels of antibiotics interfere with the expression of bacterial virulence‐associated genes depending on the type and concentration of the antibiotic used (Haddadin et al. 2010; Andersson and Hughes 2014). Therefore, we conclude that parameters that tend to reduce both bacterial populations and minimize resistance appear not to be those that result in lower virulence.

Our results provide important insights for the potential use of phage‐antibiotic combinations in controlling bacterial pathogens and pests. The strong bacterial density reduction caused by the combined action of phages and high doses of antibiotics, together with the reduced probability of bacterial resistance evolution under these conditions, support the choice of high doses of antibiotics combined with lytic phages as an evolution‐minimizing approach against bacterial pathogens and pests. We stress, however, that we only employed one phage type and that future research on other phages is needed to assess the generality of our results for this strain of *P. aeruginosa*, and that other antibiotic‐phage‐bacterial pathogen systems need to be assessed. Regulatory requirements for the employment of phages are challenging (Verbeken et al. 2014), and their use will not extend to all kinds of infection or all scenarios for a given infection type, but results such as those reported here suggest promising future research avenues for understanding and applying combined phage‐antibiotics therapies (Torres‐Barceló and Hochberg in press).

## Data archiving statement

All data can be accessed through figshare.com. doi: 10.6084/m9.figshare.2065209.

## Supporting information


**Figure S1.** Bacteria density dynamics measured every 24 h for 7 days.

**Figure S2.** Slopes of bacterial density with and without phages, for each antibiotic dose.

**Figure S3.** Synergistic effects of treatments through time: expected additive versus observed effects of combined phage‐antibiotic treatments in preventing growth in bacterial populations.

**Figure S4.** Probability of survival of *Galleria mellonella* larvae inoculated with final bacterial populations.
