# Supplementary figures and images for: Long‐term effects of single and combined introductions of antibiotics and bacteriophages on populations of Pseudomonas aeruginosa
Source: Evol Appl. 2016 Feb 18;9(4):583–95. doi: 10.1111/eva.12364 (PMC4831460; doi:10.1111/eva.12364)

# Antibiotic dose

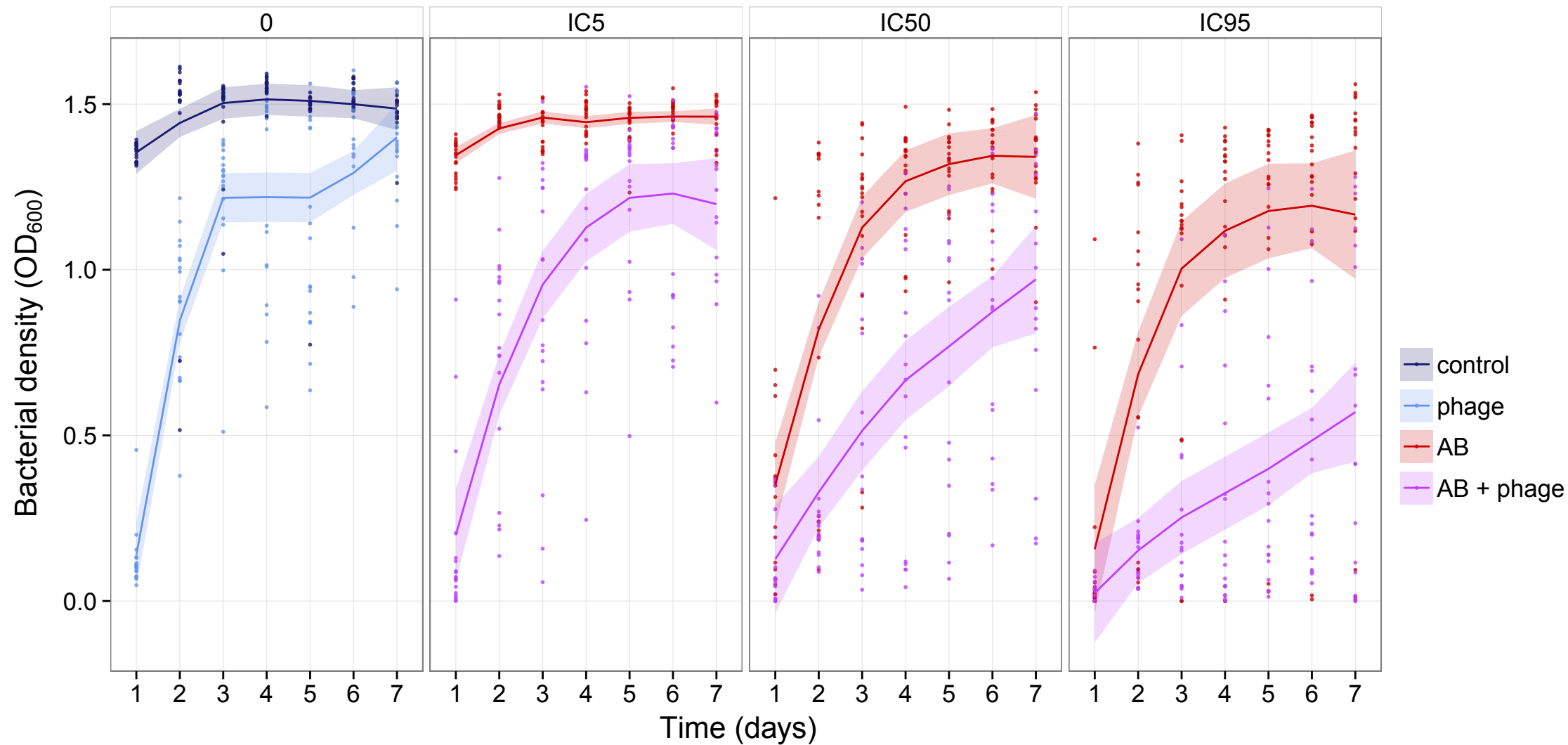

Supplement: Supplementary file 1 — Figure S1. Bacteria density dynamics measured every 24 h for 7 days. [file EVA-9-583-s001.pdf]

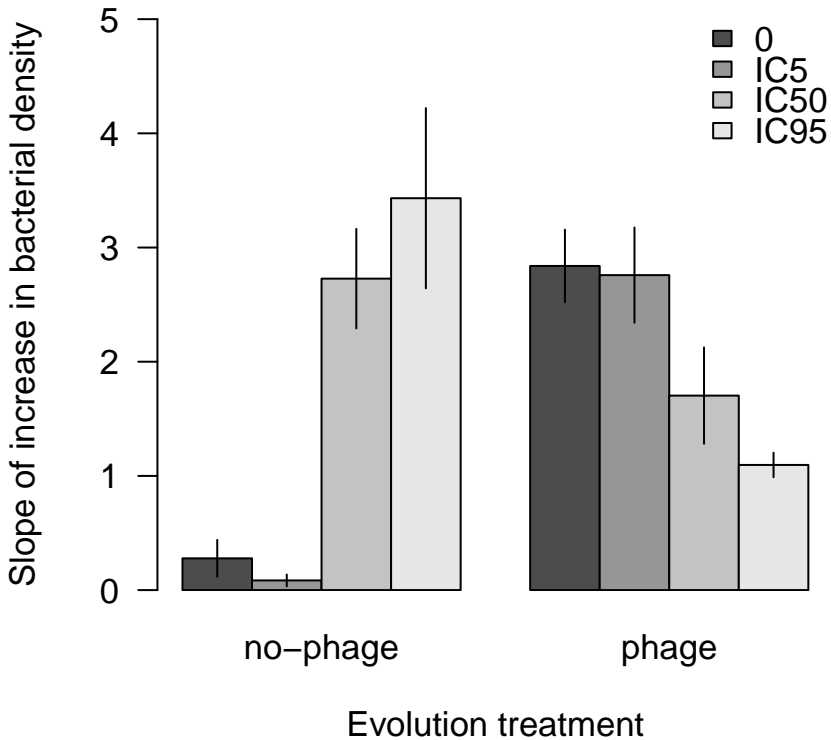

Supplement: Supplementary file 2 — Figure S2. Slopes of bacterial density with and without phages, for each antibiotic dose. [file EVA-9-583-s002.pdf]

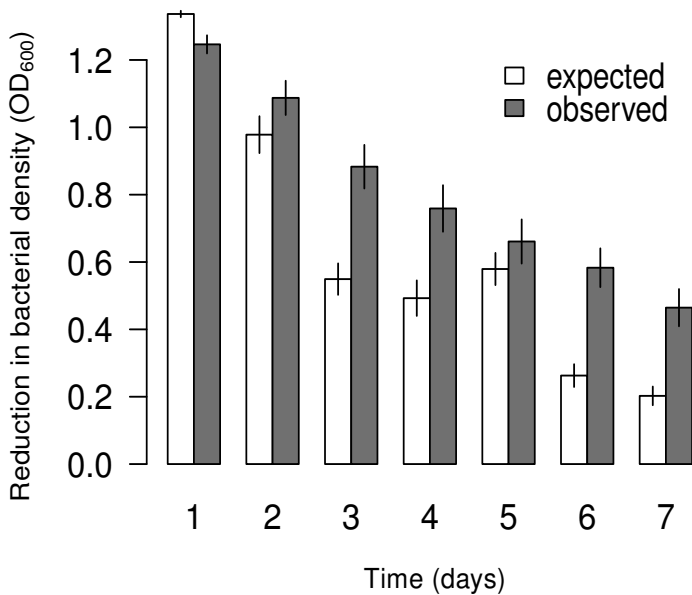

Supplement: Supplementary file 3 — Figure S3. Synergistic effects of treatments through time: expected additive versus observed effects of combined phage‐antibiotic treatments in preventing growth in bacterial populations. [file EVA-9-583-s003.pdf]

A)

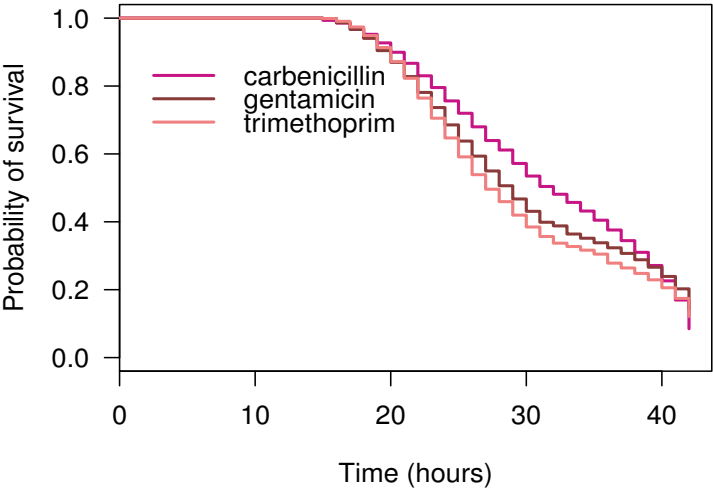

B)

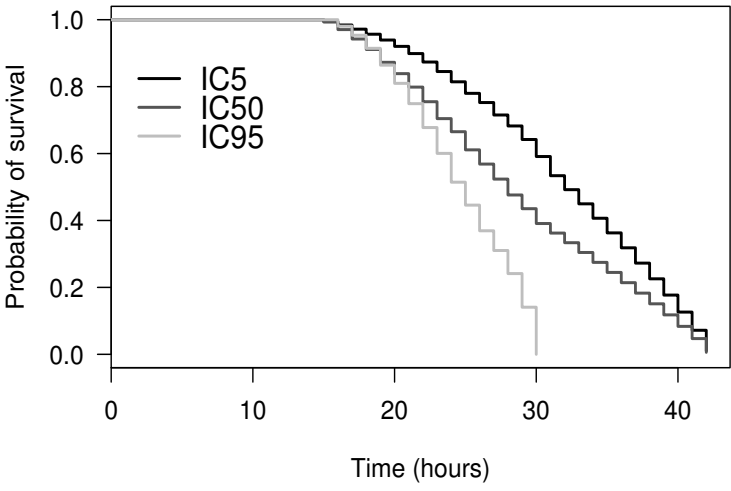

Supplement: Supplementary file 4 — Figure S4. Probability of survival of Galleria mellonella larvae inoculated with final bacterial populations. [file EVA-9-583-s004.pdf]
